# Pharmacoinformatics-enabled Interventions Improved Care Coordination and Identified Pharmacy-Related Safety Issues in a Multicultural Medicare Population

**DOI:** 10.1055/a-2297-4334

**Published:** 2024-04-24

**Authors:** Kelly J. T. Craig, Amanda L. Zaleski, Shannon M. MacKenzie, Brenda L. Butler, Rebecca A. Youngerman, Sherrie L. McNutt, Alena M. Baquet-Simpson

**Affiliations:** 1 Clinical Evidence Development, Aetna® Medical Affairs, CVS Health®, Hartford, Connecticut, United States; 2 Aetna Medicare Strategic Programs, CVS Health, New York, New York, United States; 3 Aetna Medicare Clinical Pharmacy, CVS Health, Hartford, Connecticut, United States; 4 Aetna Clinical Analytics & Behavior Change, CVS Health, New York, New York, United States; 5 Aetna Medicare Clinical Services, CVS Health, Hartford, Connecticut, United States

**Keywords:** aged, medication adherence, medication therapy management, clinical informatics, chronic disease

## Abstract

**Background** Compared to White populations, multicultural older adults experience more gaps in preventive care (e.g., vaccinations, screenings, chronic condition monitoring), social determinants of health barriers (e.g., access to care, language, transportation), and disparities and inequities (e.g., comorbidities, disease burden, and health care costs).

**Objectives** This study aims to describe an informatics-based approach used to execute and evaluate results of a member-centric, pharmacoinformatics-informed engagement program to deliver culturally tailored microinterventions to close medication-related gaps in care utilizing multidisciplinary care coordination that leverages the expanded role of the pharmacist. The operational framework will be described, and the influence of the medication use processes will be reported in a multicultural Medicare Advantage cohort.

**Methods** A pharmacoinformatics framework was leveraged to conduct a retrospective, observational cohort analysis of the program. Claims data were used to evaluate the influence of medication use process microinterventions from a large Medicare Advantage cohort of members who self-identify as Black and/or Hispanic, and have type 2 diabetes mellitus and/or hypertension, and meet eligibility criteria for multidisciplinary (e.g., nursing and pharmacy) care management (CM) and received pharmacy referral from January 1, 2022, through September 30, 2023.

**Results** A total of 3,265 Medicare Advantage members (78.3% Black and 21.7% Hispanic) received CM and pharmacy referral. Pharmacovigilance reviews conducted during this timeframe identified 258 acute events that escalated member CM. Provider outreach ( *n* = 185) informed of safety issues (drug duplication, *n* = 48; drug interactions, *n* = 21; drug–disease interactions, *n* = 5; noncompliance and/or dosing issues, *n* = 27). Outreach to members ( *n* = 160) and providers ( *n* = 164) informed of open quality-related measure gaps for medication adherence.

**Conclusion** The application of pharmacoinformatics by a payor-led multicultural clinical program demonstrated quality improvements in Medicare Advantage member identification including risk stratification, timely outreach for pharmacy-related safety issues, and improved efficiency of multidisciplinary care coordination involving medication use process workflows.

## Background and Significance

The coronavirus disease 2019 pandemic exacerbated health and health care-related disparities and inequities observed in multicultural populations. 1 2 Black and Hispanic communities, examples of historically medically marginalized populations, utilize less preventive health services in the United States when compared to White populations. 3 4 Across the gamut of preventive services, these racial and ethnic minority groups are less likely than Whites to obtain primary (e.g., influenza vaccinations), 5 secondary (e.g., colorectal screenings), 6 tertiary (e.g., chronic condition monitoring), 7 and quaternary (e.g., overmedicalization review) 1 preventive care. In addition to experiencing barriers in access to care to prevent disease and worsening of existing disease, multicultural groups have more disease burden and inferior health outcomes when compared to Whites. For instance, prevalence of chronic conditions such as type 2 diabetes mellitus (T2DM) 8 9 and hypertension is greater in Blacks and Hispanics than Whites 10 ; moreover, Black and Hispanic populations with T2DM have poorer glycemic 11 and blood pressure control, 12 than non-Hispanic White populations.

While race and ethnicity are well-known risk factors, age is also associated with disparity (i.e., differences in health closely linked with social, economic, and/or environmental disadvantage) and inequity (i.e., a type of health disparity that stems from unfair and unjust systems, policies, and practices and limits access to the opportunities and resources needed to live the healthiest life possible) in preventive health care. 13 Older adults receiving Medicare, most of whom are aged 65 years or older, have complex health needs involving several comorbidities, which require extensive preventive care. Multicultural Medicare members have more morbidity, 14 15 mortality, 16 and health care costs 16 when compared to White Medicare members. These differences necessitate the development of evidence-based interventions to decrease the disparities and inequities observed in preventive health for multicultural Medicare beneficiaries by improving the delivery of chronic disease care management (CM) for minority groups.

Pharmacotherapy is essential to chronic disease CM for Medicare members, as this population consumes more than 30% of all prescriptions. 17 Within this group, approximately 50% take ≥5 medications, and 12% take at least 10 medications regularly. 18 To help address drug therapy management in this vulnerable population, the Medicare Prescription Drug, Improvement, and Modernization Act of 2023 issued by Centers for Medicare and Medicaid Services (CMS) required that Medicare Advantage plans offering prescription drug coverage have a medication therapy management (MTM) program. 19 Plan-sponsored MTM programs are most often furnished by a pharmacist and designed to administer the plan to optimize therapeutic outcomes through improved medication use, and to reduce the risk of adverse events. The main goals of MTM programs are to (1) provide education about medication use, (2) improve medication adherence, and (3) prevent, identify, and resolve adverse drug events (ADEs). Other secondary goals of MTM programs may include improving established quality performance metrics (i.e., CMS Star Rating and Healthcare Effectiveness Data and Information Set [HEDIS] scores) and increasing member satisfaction. To achieve these goals, distinct clinical strategies are required, 20 but all necessitate member engagement and data-informed insights to address their medication use needs and to determine their eligibility for MTM; as such, a pharmacoinformatics framework must be designed, implemented, and adopted to meet the requirements to administer MTM, monitor pharmacovigilance, and improve the medication use processes for our members overall.

There is an unmet need for programs that effectively support chronic disease management in multicultural older adults. To address these needs, a payor-led Multicultural Clinical Initiative (MCI) was designed to deliver a CM program that has multiple connected goals to identify and deliver tailored preventive health interventions, including the optimization of pharmacological control of T2DM and/or hypertension in multicultural older adults and to improve medication use processes for this cohort. 21

## Objectives

This study aims to describe an informatics-based approach used to execute and evaluate results of a member-centric, pharmacoinformatics-informed engagement program to deliver culturally tailored microinterventions to close medication-related gaps in care utilizing multidisciplinary care coordination. The operational framework will be described, and the influence of the medication use processes will be reported in a multicultural Medicare Advantage cohort.

## Methods

### High-level Overview of the Multicultural Clinical Initiative Framework

The MCI framework has been previously described. 21 Briefly, several competencies were obtained to design an intervention appropriate for this population: (1) patient-centered (e.g., care focuses more on the patient's problem than the diagnosis), (2) transdisciplinary (e.g., inclusive care team beyond clinicians including social and societal services), (3) evidence-based (e.g., use of best available evidence to support decision-making), (4) quality improvement-oriented (e.g., the systematic improvement of care), and (5) informatics-enabled (i.e., the integration of digital technology to transform data into insights that can be acted upon).

Implementation of this culturally tailored engagement and its interventions were informed by core competencies listed above. During intervention mapping, competencies obtained were paired with evidence-based frameworks to support health equity advancement, particularly in areas related to social determinants of health (SDoH) and health behavior. These formative studies supported the phase-based clinical CM. Three separate prospective qualitative interviews were conducted to better understand the health and health care needs of a multicultural Medicare member population. The interviews were conducted with follow-up quantitative surveys and content analysis in the following groups: (1) Black and/or Hispanic Medicare consumers, (2) providers (i.e., the care team) treating this population, and (3) health plan colleagues (i.e., case managers and social workers employed by the large health plan). 21

### Overview of the Multicultural Clinical Initiative Pharmacoinformatics Framework

**Fig. 1:**
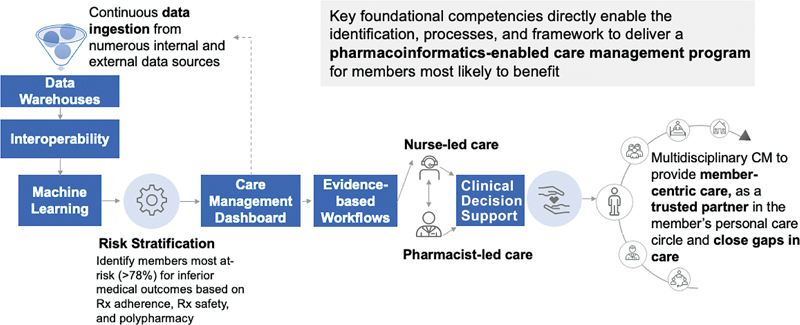
Multicultural Clinical Initiative pharmacoinformatics framework. Key foundational competencies directly enable the identification and delivery of culturally tailored microinterventions to close medication-related gaps (e.g., medication adherence, statin use in persons with T2DM) in care utilizing multidisciplinary care coordination. CM, care management; Rx, prescription; T2DM, type 2 diabetes mellitus.

The MCI was operationalized across eight key components ( Fig. 1 ): (1) a rich and diverse data foundation; (2) application of artificial intelligence techniques; (3) interoperability processes between data warehouses containing medical and pharmacy claims data, health care utilization, member data summaries, and electronic health records; (4) multiple data platforms curating member-specific HEDIS and CMS Star Ratings measures to inform gaps in care; (5) CM engagement dashboard for the MCI population featuring logic-queried Star- and HEDIS-driven clinical intervention factors specific to pharmacy measures; (6) evidence-based nursing- and pharmacy-related standardized workflows; (7) utilization of clinical decision support (CDS) tools; (8) a designated platform for nurse care managers and pharmacists with cultural competency training to deliver information and knowledge using multichannel for member and provider engagement. The expanded scope of pharmacists' practice in this program enables clinical preventive services, chronic disease management, and transitions of care to address unmet clinical and social needs of this population including health inequities and health disparities.

### Risk-stratified Member Identification

The MCI program leverages interoperability between data warehouses for risk stratification of member eligibility. Rule-based logic and predictive modeling identify Medicare Advantage members that self-identify as Black and/or Hispanic with diagnoses of T2DM and/or hypertension, residing in Texas (TX), Florida (FL), or Pennsylvania (PA), and an a priori population health risk stratification score threshold of  > 78%. These states (i.e., TX, FL, and PA) were selected based on member need (i.e., high prevalence of Black and Hispanic members and/or disparities in gaps in care). The risk stratification algorithm (U.S. patent pending) is an evidence-based, proprietary scoring tool that leverages seven different predictive models to estimate clinical and economic burden and actionable metrics (i.e., fall risk, drug safety indications, and avoidable emergency department visits). Predictive models are based on a plurality of data sources such as demographics, medical and pharmacy claims, diagnosis codes, biomarkers, laboratory results, health care utilization, gaps in care, and SDoH. The inclusion threshold (i.e., greatest risk for inferior medical outcomes > 78%) is based on member risk, potential benefit, and care manager capacity.

All eligible members are routed to a nurse-led CM program following standardized workflows supporting CMS-required MTM including pharmacist-led consultation, if eligible. Event-driven alerts, such as an inpatient stay, escalate intensity of CM activities such as decreased time to CM outreach and provision of synchronous medication review and consultation.

### Dashboard for Care Management Engagement

Warehouse data are operationalized on a dashboard for nurse-led CM and quality measures specific to HEDIS and CMS Star Ratings are leveraged. These quality metrics are well-established and widely used evidence-based tools to evaluate health care quality and performance. As such, a majority of health plans and health care organizations are required to report these data on an annual basis. Their widespread adoption in the health care industry renders them ideal for comparative analysis and program benchmarking. Specific to this study, these data inform if medication-related gaps in care (e.g., received statin therapy, poor glycemic control [hemoglobin A1c > 9%]) are met. Additionally, those measures (e.g., related to Rx nonadherence) are utilized to derive clinical intervention factors indicating members are at-risk of suboptimal medication-related outcomes along with other proprietary algorithms to monitor for drug safety (e.g., interactions, contraindications) and polypharmacy (e.g., number of drugs prescribed per member).

### Nurse-led Care Pathway

Upon eligibility, CM is notified of engagement prioritization and members are outreached telephonically by a dedicated nurse CM by order of priority. Prior to CM delivery, the CM (1) conducts a medical chart review via an internal CM platform with data warehouse interoperability; (2) consults quality dashboards to review medication-related gaps in care and risks; (3) performs standardized workflows to complete a thorough medication review including medication reconciliation; (4) escalates and/or facilitates communication with the provider(s) and dispensing pharmacy of record. The care manager utilizes documentation templates for case management and care planning including SDoH care considerations. All MCI care managers hold an active, unrestricted registered nurse license and receive specialized competency training following a structured, evidence-based curriculum. The expanded skill set enables care managers to develop trusting, meaningful, and mutually beneficial relationships with the member and their care team. After foundational trust is established, care managers provide ongoing, support, education, and assistance to members through various channels (i.e., telephonic, email, and mail). CM activities vary depending on gaps in care; however, general goals of CM engagement are to (1) provide support and education for members; (2) deploy strategies to improve medication adherence; (3) reinforce behavioral strategies for chronic condition management; (4) complete and document standardized assessments; (5) facilitate communication and/or referrals with other members of the care team, if necessary. All members are then referred to a dedicated pharmacist for additional care coordination.

### Pharmacist-led Care Pathway

A dedicated pharmacist (1) conducts a comprehensive medication review or MTM, if eligible, utilizing the claims data warehouse; (2) outreaches members' providers to remedy medication-related concerns (e.g., duplicative treatments, dose optimization, gaps in care, and high-risk medications); (3) uses CDS tools to review potential medication interactions, contraindications, and legacy medications; (4) provides medication adherence counseling to explore barriers (i.e., accessibility and/or resources) and map members to resources available within their benefit plan and geography (e.g., autorefills, 100-day maximum supply, lowest cost pharmacy). Members with a recent hospital discharge to home are escalated using event-driven alerts, prioritized for urgent case review (i.e., postdischarge medication reconciliation), and are provided with transitions of care from a dedicated team. At the close of the consultation, the pharmacist will provide the member and provider with documentation including the MTM review, if applicable.

### Study Design Overview

The pharmacoinformatics framework was leveraged to conduct a retrospective, observational cohort analysis of the MCI program. Administrative claims data were de-identified, aggregated, and analyzed to evaluate the influence of the medication use processes microinterventions conducted January 1, 2022, through September 30, 2023. Inclusion criteria for the study were Medicare Advantage members with intervention eligibility who identify as Black and/or Hispanic with diagnoses of hypertension and/or T2DM; residing in FL, TX, or PA; meeting a risk stratification score threshold (> 78th percentile); and received a pharmacy referral. Exclusion criteria consisted of members with recent CM activity in the last 60 days and/or engaged in or enrolled in CM in the last 90 days; specific plans (dual eligible special needs or value-based plans); members with diagnoses of rare diseases, chronic kidney disease, and/or heart failure, and members enrolled in specialty clinical CM including cancer programs or value-based design programs; and receipt of hospice care or recent stay at a long-term care facility. Primary outcomes were pharmacy engagement metrics, such as total referrals, and pharmacovigilance to identify acute events to escalate CM and other safety-related measures including drug duplication, drug interactions, drug–disease interactions, noncompliance, and dosing issues. Secondary outcomes were member and provider outreach as a proxy for informatics-identified quality-related gaps in medication adherence.

## Results

A total of 3,721 Medicare Advantage members (78.3% Black and 21.7% Hispanic) were engaged in MCI CM and received pharmacy referral from January 1, 2022, through September 30, 2023. Of these engaged members, 95% had a physician relationship and 54% had four or more chronic diseases. During this timeframe, pharmacist referral and consultation identified 258 acute events that escalated their CM. Additionally, pharmacy reviews with provider outreach ( *n* = 185) informed polypharmacy-related safety issues including drug duplication ( *n* = 48), drug interactions ( *n* = 21), drug–disease interaction ( *n* = 5), and noncompliance and/or dosing issues ( *n* = 27). Outreach ( *n* = 160) was provided to inform members of gaps in care for specific Stars and HEDIS measures related to medication adherence. Provider outreach was delivered to address Stars-informed care gaps related to statin use in persons with diabetes ( *n* = 80) and management of chronic obstructive pulmonary disease ( *n* = 6) as a comorbidity.

## Discussion

Medication dispensing is the best-known role of the pharmacist, but pharmacists are a key member of the health care team and scope of practice extends beyond that responsibility. Health care reform law, such as the Patient Protection and Affordable Care Act, 22 supports the expanded scope of work for pharmacists to provide counseling, MTM, and disease state management, for example, which reimburses these activities. 23 In this study, the expanded role of the pharmacists' patient care process 24 enables care coordination with CM nurses and other providers (e.g., member's primary clinician) to optimize and personalize member health and medication-related outcomes in a multicultural senior population to alleviate health disparities and advance health equity. The patient-centeredness approach, whereby engagement between the pharmacist and the Medicare members, has demonstrated improved medication adherence. 25

The employment of informatics-based operational tools to provide actionable insights was leveraged to identify needs and improve medication use-related workflows in Medicare members led by a multidisciplinary team. To identify Black and or/Hispanic members most at-risk for poor clinical outcomes, a proprietary population-based health risk score that incorporated various predicted risks and effect on care outcomes was calculated for each member. Operational key performance indicators (e.g., number of enrollees and engagement rate) and clinical outcomes (e.g., monthly performance rates for quality measures) were monitored monthly using a dashboard. Real-time analytical and artificial intelligence-based tools were leveraged to provide timely information to prepare tailored pharmacy-related microinterventions led by the pharmacist, including pharmacovigilance monitoring.

Pharmacists play a pivotal role whereby they maintain the rational and safe use of medicines; they are engaged in pharmacovigilance activities including identification of acute events and management of episodic ADEs to support medication and patient safety. ADEs are responsible for 15% of hospital admissions in patients 65 years or older and 20% of patients admitted to intensive care. 26 27 Addressing risk factors (e.g., polypharmacy, comorbidities, length of hospital stay, cardiovascular agents, anti-infection treatments) 28 29 for ADEs in an outpatient setting is challenging, but optimizing informatics-driven workflows provides population-level insights at scale, but drill down to identify and mitigate individual acute (e.g., severe ADEs) and chronic (e.g., medication compliance) risks. This study identified 258 acute events that triggered escalated CM. These findings have significant real-world implications as they highlight the critical role of *actionable* , informatics-enabled interventions that successfully prevent avoidable disease exacerbation, complications, and downstream sequalae.

This payor-led pharmacoinformatics framework supports the timely resolution of pharmacy-related risks for Medicare members by enhanced communication. Unlike traditional communications between the pharmacists and prescriber that are transaction-based and single-drug-focused at the point of dispensing, the pharmacist-led surveillance system coordinates communication with the member, and their nurse care manager and provider/prescriber into a process of care that is ongoing and person-centered. 30 Further, contrary to most pharmacist–prescriber interactions, the MCI program pharmacist has access to relevant health care data with expanded interoperability into warehouses (e.g., electronic health records (EHR), medical and pharmacy claims) to derive a complete picture of the member's health (e.g., medical history, all medications being taken, diagnostic findings to target correct therapies, etc.) at the point of care and can surmount Health Insurance Portability and Accountability Act (HIPAA)-related barriers, as the member's health care benefits are covered by the payor. 30 Management of chronic conditions in these high-risk Medicare members requires coordination of information to bring resolution. In addition to clinical data, pharmacists collect information from the member to identify and deliver education needs, and assess barriers and behaviors, while acting as a trust broker of their care. To foster that trust and build the relationship, the multidisciplinary team has received cultural competency training and employed motivational interviewing techniques to better understand members' health status and foster deeper relationships with their clinicians by their ongoing and person-centered communications.

This study has a few limitations. Currently, the program is only offered to eligible members who self-report as Black and/or Hispanic and reside in FL, TX, or PA, as such the sociodemographic scope and geography of the study are limited. Additional members may be eligible for the program, as race and ethnicity reporting are often incomplete demographic fields in structured health care data. Efforts to improve race and ethnicity data collection will enhance the ability to provide more services; moreover, in an increasingly diverse multiracial and multiethnic world, representation is critical and there is a moral imperative to ensure datasets are accurate and inclusive. However, socioracial asymmetries persist, such that individuals of historically marginalized races/ethnicities, and mixed races and/or ethnicities do not self-report due to experienced social justice issues including racism. At the time of submission, data for the HEDIS Measuring Year 2023 were not available. Therefore, the data presented only fully represent 2022 HEDIS-related member outcomes attributable to program microinterventions. Additionally, future mixed-methods studies will include qualitative data to evaluate member, provider, and care manager experiences to corroborate program outcomes and directly inform program enhancement and expansion.

The MCI program will continue to invest in its organizational capacities to further enable interoperability in the multicultural data ecosystem (e.g., gaps in care, utilization, patient safety) as additional member-level race, ethnicity, and language data and cohort-level demographic and geographic data sets are acquired, assimilated, and aggregated. Data processing and additional analytics-driven modeling will support knowledge sharing between data assets to facilitate real-time notifications to improve decision support and timely member outreach that influences member experience. Additionally, the expansion of CDS tools for CM providers has the potential to increase efficiency and streamline pharmacy-related workflows for member engagement.

In the future, the MCI program will expand to include all geographies and other populations (e.g., Asian, Native American) of Medicare members and broaden community engagement. Risk stratification thresholds and their corresponding quality measures will pinpoint additional geographic hotspots of need, and the expansion of local and community partnerships to provide education, resources, and support for custom events (e.g., heart and mental health) will be provided to address the identified disparities. Organizations can better support Medicare members by broadening engagement and connectivity, both operationally with resources and needs assessment, beyond clinical settings; partnerships between the providers and the communities where they serve will support community-based preventive interventions to provide vaccinations, health screenings, and education.

## Conclusion

The application of pharmacoinformatics by a payor-led MCI program demonstrated quality improvements in Medicare Advantage member identification including risk stratification, timely outreach for pharmacy-related safety issues, and improved efficiency of multidisciplinary care coordination involving medication use process workflows.

## Clinical Relevance Statement

Informatics-driven improvement of medication use processes led by payors can positively augment health and health equity outcomes in older multicultural populations at scale using a multidisciplinary CM program based on evidence-based practice and inclusion of cultural competencies. As our health care system prepares for a rapidly aging population that is becoming increasingly racially and ethnically diverse, it underscores the importance of tailoring health care interventions to meet the specific needs of our nation's population.

## Multiple Choice Questions

1. What are the goals of MTM?
  - Provide education about medication use
  - Improve medication adherence
  - Prevent, identify, and resolve ADEs
  - All of the above are goals of MTM.
  Correct answer: d. MTM services are intended to address issues of polypharmacy, preventable ADEs, medication adherence, and medication misuse.
2. Pharmacovigilance is the science and activities related to the detection, assessment, understanding, and prevention of adverse effects or any other medicine-related problem. What are examples of safety-related measures observed for pharmacovigilance?
  - Drug duplication
  - Drug interactions
  - Drug–disease interactions
  - Dosing issues
  - All of the above are example of safety-related measures for pharmacovigilance.
  Correct answer: e. Pharmacovigilance should improve patient care and safety related to use of medicines, as such, data surveillance within pharmacy information systems to identify safety-issues related within the medication use processes step of monitoring and reporting is critical. Safety-related measures include identification of drug duplication, drug interactions, drug–disease interactions, polypharmacy, and dosing issues.
